# Predictors of *Trypanosoma cruzi* PCR positivity in patients with chronic Chagas disease

**DOI:** 10.1590/0074-02760230115

**Published:** 2023-12-15

**Authors:** Ana Carolina Bastos de Lima, Veronica Gonçalves Mendes, Roberto Rodrigues Ferreira, Lindice Mitie Nisimura, Samuel Iwao Maia Horita, Henrique H Veloso, Andréa R Costa, Gilberto Marcelo S da Silva, Luiz Henrique C Sangenis, Marcelo T Holanda, Lorena Rimolo, Ademir B Cunha, Luciana Ribeiro Garzoni, Alejandro Marcel Hasslocher-Moreno, Mauro Felippe F Mediano, Otacílio da Cruz Moreira, Constança Britto, Roberto M Saraiva

**Affiliations:** 1 Fundação Oswaldo Cruz-Fiocruz, Instituto Oswaldo Cruz, Laboratório de Biologia Molecular e Doenças Endêmicas, Rio de Janeiro, RJ, Brasil; 2 Fundação Oswaldo Cruz-Fiocruz, Instituto Nacional de Infectologia Evandro Chagas, Laboratório de Pesquisa Clínica em Doença de Chagas, Rio de Janeiro, RJ, Brasil; 3 Fundação Oswaldo Cruz-Fiocruz, Instituto Oswaldo Cruz, Laboratório de Inovações em Terapias, Ensino e Bioprodutos, Rio de Janeiro, RJ, Brasil; 4 Universidade Federal Fluminense, Hospital Universitário Antonio Pedro, Niterói, RJ, Brasil; 5 Fundação Oswaldo Cruz-Fiocruz, Instituto Oswaldo Cruz, Laboratório de Virologia e Parasitologia Molecular, Rio de Janeiro, RJ, Brasil

**Keywords:** Chagas disease, Trypanosoma cruzi, clinical forms, biomarkers

## Abstract

**BACKGROUND:**

A positive *Trypanosoma cruzi* polymerase chain reaction (PCR) is associated with a worse prognosis in patients with chronic Chagas disease (CD).

**OBJECTIVES:**

To study the association of clinical, electrocardiographic, and echocardiographic characteristics and biomarker blood levels with positive *T. cruzi* PCR in chronic CD.

**METHODS:**

This is a single-centre observational cross-sectional study. Positive *T. cruzi* PCR association with clinical, electrocardiographic, and echocardiographic characteristics, and biomarker blood levels were studied by logistic regression analysis. p values *<* 0.05 were considered significant.

**FINDINGS:**

Among 333 patients with chronic CD (56.4% men; 62 ± 10 years), *T. cruzi* PCR was positive in 41.1%. Stepwise multivariate logistic regression showed an independent association between positive *T. cruzi* PCR and diabetes mellitus {odds ratio (OR) 0.53 [95% confidence interval (CI) 0.30-0.93]; p = 0.03}, right bundle branch block [OR 1.78 (95% CI 1.09-2.89); p = 0.02], and history of trypanocidal treatment [OR 0.13 (95% CI 0.04-0.38); p = 0.0002]. Among patients with a history of trypanocidal treatment (n = 39), only four (10%) patients had a positive *T. cruzi* PCR.

**MAIN CONCLUSIONS:**

Among several studied parameters, only diabetes mellitus, right bundle branch block, and history of trypanocidal treatment showed an independent association with positive *T. cruzi* PCR. History of trypanocidal treatment was a strong protective factor against a positive *T. cruzi* PCR.

Chagas disease (CD) is a neglected tropical disease caused by the *Trypanosoma cruzi* parasite and affects 5-7 million people, mainly in low-income populations in endemic areas of Latin America, where the main mode of transmission is vector-borne.^1^ However, migration has spread the disease to non-endemic countries where other forms of transmission, such as blood transfusion, organ donation, and vertical transmission, may occur. Those countries include but are not limited to the USA, with an estimated 288,000 infected individuals,^2^ and European countries, with an estimated 120,000 infected individuals.^3^ Therefore, CD is still a challenge for public health, causing more than 7,000 deaths per year^4^ and a high economic and health burden.^5^ ^,^ ^6^ ^,^ ^7^

Chronic CD can have three different clinical forms, ranging from a long-lasting indeterminate form, without clinical evidence of a major organ damage, to cardiac and digestive forms, which can occur simultaneously. Patients with the cardiac form may present a dismal prognosis due to arrhythmias, thromboembolic events, and heart failure (HF).^8^ However, the mechanisms underlying the progression from the indeterminate to the cardiac form comprehend several possible pathways that are still under investigation. For decades, it was believed that the parasite was not present in the chronic phase and that auto-immune mechanisms would prevail and determine the progression of the disease.^9^ However, CD reactivation in immunocompromised patients and molecular studies proved otherwise.^10^ ^,^ ^11^ Today, the persistence of the parasite seems to be a key element for the persistent low grade chronic fibrosing myocarditis present in chronic CD.^1^ ^,^ ^12^ ^,^ ^13^ Moreover, an imbalance between pro- and anti-inflammatory cytokines is regarded as an important element in CD progression.^12^ ^,^ ^13^

The importance of the persistence of the parasite for CD progression was suggested by several findings, such as a more frequent identification of the parasite in the bloodstream of patients with the cardiac form than in those without the cardiac form,^14^ ^,^ ^15^ and a worse prognosis in patients with parasitaemia identified by haemoculture^15^ and molecular methods.^16^ ^,^ ^17^

We therefore aimed to investigate which clinical characteristics and immunological biomarkers would correlate with a positive *T. cruzi* polymerase chain reaction (PCR) in patients with chronic CD.

## SUBJECTS AND METHODS

*Design and study subjects* - This is a cross-sectional secondary analysis of a previous study approved by the local ethical committee on 01/27/2014 under number 515851 whose main paper was already published.^17^

Data from adult patients with chronic CD who had been included in the previous study between July 2014 and March 2017 and who had had blood samples collected for *T. cruzi* quantitative PCR (qPCR) were analysed in the present paper.^17^ Briefly, from 402 patients, 11 did not return for the index echocardiogram, 30 were excluded due to several reasons described in Figure, and other 28 patients did not have blood samples collected for *T. cruzi* qPCR. The final studied population was composed of 333 patients (Figure).

**Figure:**
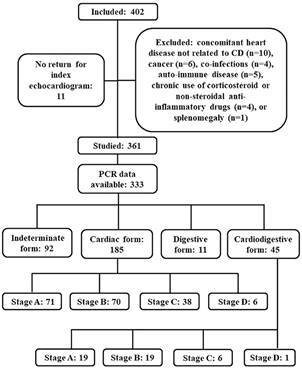
Flow diagram depicting recruitment, exclusion criteria, and studied groups at baseline.

CD diagnosis was based on positivity in two different serological tests (enzyme-linked immunosorbent assay and indirect immunofluorescence).^18^

Data regarding clinical characteristics, comorbidities, self-reported skin colour, previous trypanocidal treatment history, and use of medications were based on face-to-face interviews complemented by medical records review. Skin colour was categorised into white, brown, black, yellow, and indigenous, following the classification used by the Brazilian Official Bureau of Statistics (Instituto Brasileiro de Geografia e Estatística - IBGE), changing only brown for mixed-race.^19^ As no patient self-reported as yellow, only four categories were described in Table I.

**TABLE I:** Clinical and echocardiographic characteristics of studied subjects (n = 333)

Variable	
Chagas disease clinical forms	
Indeterminate	92 (27.6)
Digestive	11 (3.3)
Cardiac	185 (55.5)
Stage A	71 (21.3)
Stage B	70 (21.0)
Stage C	38 (11.4)
Stage D	6 (1.8)
Cardiodigestive	45 (13.5)
Stage A	19 (5.7)
Stage B	19 (5.7)
Stage C	6 (1.8)
Stage D	1 (0.3)
Age, years	61.7 ± 10.4
Sex, men	188 (56.4)
Body mass index, kg/m^2^	27.3 ± 5.1
Arterial hypertension	228 (68.5)
Diabetes mellitus	76 (22.8)
Previous trypanocidal treatment	39 (11.7)
Self-reported skin colour	
White	79 (23.7)
Black	45 (13.5)
Indigenous	10 (3.0)
Mixed-race	199 (59.8)
Electrocardiogram	
RBBB	116 (34.8)
LBBB	14 (4.2)
LAHB	100 (30.0)
Primary T wave changes	102 (30.6)
Electric inactive areas	29 (8.7)
Low voltage	24 (7.2)
Cardiac device	44 (13.2)
2D echocardiogram	
LA volume, ml/m^2^	34.0 ± 14.8
LVd, cm	5.5 ± 0.8
LVs, cm	3.6 ± 1.2
LV ejection fraction, %	58.4 ± 13.7
E/E’ ratio	10.4 ± 4.9
LV aneurysm	50 (13.8)
LV systolic function	
Normal	248 (74.5)
Mild dysfunction	47 (14.1)
Moderate dysfunction	23 (6.9)
Severe dysfunction	15 (4.5)
LV diastolic dysfunction	
Normal	98 (29.4)
Grade I	156 (46.8)
Grade II	46 (13.8)
Grade III	17 (5.1)
Non-classifiable	16 (4.8)
Medications	
Carvedilol	87 (26.1)
ACE inhibitors	143 (42.9)
ARB	105 (31.5)
Digoxin	20 (6.0)
Spironolactone	42 (12.6)
Furosemide	49 (14.7)
Warfarin	43 (12.9)
Amiodarone	38 (11.4)
Metformin	66 (19.8)

ACE: angiotensin converting enzyme; ARB: angiotensin receptor blocker; E: peak early wave diastolic filling velocity; E’: peak early diastolic mitral annulus velocity; LA: left atrial; LAHB: left anterior haemiblock; LBBB: left bundle branch block; LV: left ventricular; LVd: LV end-diastolic diameter; LVs: LV end-systolic diameter; RBBB: right bundle branch block. Values are mean ± standard deviation (SD) or n (%).

Patients were classified at the time of their enrolment in the study according to the Brazilian CD consensus^9^ into: indeterminate (no evidence of cardiac involvement), stage A [no HF symptoms with isolated changes in the electrocardiogram (ECG)], stage B (no HF symptoms with segmental or global LV systolic dysfunction), stage C (symptomatic HF), or stage D (end-stage HF). ECGs were analysed by experienced cardiologists and classified using the Minnesota Code criteria, modified for CD.^20^ The ECG changes considered compatible with CD cardiac form followed previously published criteria.^9^

*Echocardiography* - Studies were performed using a phased-array ultrasound system (Vivid 7, GE Medical Systems, Milwaukee, WI) equipped with M4S phased-array and 1.5- to 4-MHz four-matrix-array transducers by a single experienced echocardiographer. Cardiac dimensions, measured using M-mode and 2D echocardiography, and Doppler measurements were obtained as recommended.^21^ ^,^ ^22^ 2D LV ejection fraction and 2D maximum LA volume were determined using modified Simpson’s rule with images obtained from apical 4- and 2-chamber views.

*Biomarkers measurement* - Biomarker blood measurements were performed by researchers blinded to the clinical classification of the patients. Tissue inhibitor of metalloproteinase-1 (TIMP-1), TIMP-4, interleukin-6 (IL-6), IL-10, and IL-17 measurements were performed using commercially available enzyme-linked immunosorbent assay (ELISA) kits (TIMP-1, and TIMP-4: Picokine ELISA Human ImmunoAssay, Boster Biologics, CA, USA; IL-6, IL-10, and IL-17: Quantikine ELISA Human ImmunoAssay, R&D systems, EUA) according to the manufacturer’s instructions using SpectraMax 190 absorbance micro plate reader (Molecular Devices, CA, USA).

*Trypanosoma cruzi PCR* - *T. cruzi* PCR was performed as previously described.^17^ Briefly, ten millilitres of whole blood samples were obtained from each patient. The samples were processed for DNA extraction using the High Pure PCR Template Preparation kit (Roche Diagnostics Corp., Indianapolis, IN). The qPCR assays were performed by absolute quantification to estimate the parasite load, as previously described.^23^ The qPCR assays were carried out with 5 *μL* of DNA, using the FastStart Universal Probe Master Mix (Roche Diagnostics, Mannheim, Germany) in a final volume of 20 *μL*. A sample was considered positive when the fluorescence generated by the Cruzi 3 TaqMan probe (FAM) crossed the threshold set at 0.02 for the *T. cruzi* satellite DNA target, at least in one of the technical replicates, during the 40 cycles of the real time PCR assay. The amplifications were performed in an ABI 7500 Fast Real Time PCR device (Applied Biosystems, USA).

*Statistical analysis* - Calculations were done using MedCalc version 20.113 (MedCalc Software, Mariakerke, Belgium) and Stata version 13.0 (StataCorp, College Station, TX). Continuous variables are expressed as mean ± standard deviation or median (interquartile range), and categorical variables as absolute and percentage values. Kolmogorov-Smirnov tests provided support that continuous variables were normal, as p values were > 0.10. Data between groups were compared using one-way analysis of variance (ANOVA) followed by Student-Newman-Keuls post-hoc analysis, Kruskal-Wallis test followed by Dunn’s multiple comparison test, or contingency tables, as appropriate. Associations between studied variables and *T. cruzi* PCR positivity were tested using univariate logistic regression analysis. All variables with a p value under 0.20 were entered in a stepwise multivariate logistic regression analysis. Associations between continuous variables and parasite load were tested using linear regression after parasite load log transformation due to its skewed distribution. Missing data were handled by listwise deletion. The null hypothesis was rejected at p *<* 0.05. The data that support the findings of this study are available on request from the corresponding author.

*Ethics* - This study was approved by the local ethical committee on 01/27/2014 under number 515851. This study conformed to standards currently applied by the Brazilian National Committee for Research Ethics and Resolution 466/2012 of the National Health Council, of the Ministry of Health, and to the Declaration of Helsinki of 1975, as revised in 1983. All subjects gave their written informed consent before participating.

## RESULTS

*Patients*- The studied patients presented the following CD clinical forms at the time of their enrolment: 92 (27.6%) presented the indeterminate form, 185 (55.6%) presented the cardiac form [stage A (n = 71; 21.3%), stage B (n = 70; 21.0%), stage C (n = 38; 11.4%), stage D (n = 6; 1.8%)], 11 (3.3%) presented digestive form, and 45 (13.5%) presented cardiodigestive form [stage A (n = 19; 5.7%), stage B (n = 19; 5.7%), stage C (n = 6; 1.8%), stage D (n = 1; 0.3%)].

The clinical characteristics, laboratory results, electrocardiogram and echocardiogram findings of the included patients are depicted in Table I. The most common ECG abnormalities were right bundle branch block, left anterior haemiblock, and primary T wave changes. Most patients presented a normal LV systolic function and grade I LV diastolic dysfunction (Table I). Forty-four patients (13.2%) had a cardiac device at baseline: 24 (7.2%) had a dual-chamber pacemaker, seven (2.1%) had a single-chamber pacemaker, eight (2.4%) had an implantable cardioverter-defibrillator (ICD) implant, 3 (0.9%) had a cardiac resynchronisation therapy pacemaker device, and 2 (0.6%) had a cardiac resynchronisation therapy defibrillator device. The frequency of use of cardiovascular medications was proportional to the number of patients with more advanced stages of the cardiac form and/or concomitant arterial hypertension.

A total of 39 patients had a previous history of trypanocidal treatment. The mean time elapsed between trypanocidal treatment and blood collection for *T. cruzi* PCR was 11.1 ± 7.2 years. Twenty-four patients (61.5%) received at least 300 mg a day of benznidazole. Other 14 patients were also treated with benznidazole: one patient (2.6%) received 100 mg a day, nine patients (23.1%) received 200 mg a day, and in four patients (10.3%) the dose information was missing. An additional patient was treated with ketoconazole. The trypanocidal duration of treatment was at least 60 days in 33 patients (84.6%), less than 60 days in four patients (10.3%), and unknown in one patient (2.6%).

*Trypanosoma cruzi PCR positivity* - PCR for *T. cruzi* DNA was positive in 137 out of 333 (41.1%) patients. *T. cruzi* PCR positivity was similar across the studied groups: 34.8% in the indeterminate group; 42.4% in stages A/B of the cardiac form; 47.1% in stages C/D of the cardiac form; and 45.4% among patients with the digestive form (p = 0.48).

*Univariate analysis between T. cruzi PCR positivity and studied variables* - The only clinical characteristic associated, in the univariate analysis, with a positive *T. cruzi* PCR was a previous history of trypanocidal treatment, which was strongly protective. The only ECG characteristic associated with *T. cruzi* PCR result was the presence of a right bundle branch block that increased by 65% the chance of a positive *T. cruzi* PCR. There was no 2D echocardiographic parameter associated with a positive *T. cruzi* PCR (Table II).

**TABLE II:** Univariate associations between clinical, echocardiographic, and biomarkers characteristics of studied subjects and *Trypanosoma cruzi* polymerase chain reaction (PCR) positivity

Variable	OR	95% CI	p value	AUC (95% CI)
Cardiac form	1.37	0.85-2.22	0.19	0.53 (0.48-0.59)
Age, years	1.00	0.98-1.02	0.85	0.51 (0.45-0.56)
Sex, men	0.70	0.45-1.09	0.11	0.54 (0.49-0.60)
Body mass index, kg/m^2^	0.96	0.92-1.00	0.06	0.58 (0.52-0.63)
Hypertension	1.35	0.84-2.18	0.21	0.53 (0.48-0.59)
Diabetes mellitus	0.63	0.37-1.09	0.10	0.54 (0.48-0.59)
Previous trypanocidal treatment	0.14	0.05-0.40	<0.0001	0.57 (0.52-0.63)
Electrocardiogram				
RBBB	1.65	1.05-2.61	0.03	0.56 (0.50-0.61)
LBBB	1.08	0.36-3.17	0.89	0.50 (0.45-0.56)
LAHB	1.11	0.69-1.79	0.65	0.51 (0.46-0.57)
Primary T wave changes	0.89	0.55-1.43	0.63	0.51 (0.46-0.57)
Electric inactive areas	1.60	0.74-3.43	0.23	0.51 (0.46-0.57)
Low voltage	1.23	0.53-2.83	0.63	0.51 (0.45-0.56)
Cardiac device	1.10	0.58-2.09	0.77	0.51 (0.45-0.56)
2D echocardiogram				
LA volume, ml/m^2^	1.00	0.99-1.01	0.95	0.51 (0.45-0.56)
LVd, cm	0.89	0.68-1.18	0.43	0.52 (0.47-0.58)
LVs, cm	1.00	0.83-1.21	0.98	0.51 (0.45-0.56)
LV ejection fraction, %	1.00	0.98-1.01	0.81	0.53 (0.47-0.58)
E/E’ ratio	1.01	0.97-1.06	0.65	0.50 (0.45-0.56)
LV aneurysm	0.63	0.33-1.19	0.15	0.53 (0.47-0.58)
Biomarkers				
BNP, ng/mL	1.00	0.99-1.00	0.62	0.50 (0.44-0.56)
Troponin I, ng/mL	0.16	0.01-9.58	0.17	0.54 (0.48-0.59)
TGF-β1, ng/mL	1.00	0.99-1.02	0.54	0.52 (0.46-0.58)
TNF, pg/mL	1.02	0.96-1.08	0.53	0.54 (0.48-0.60)
IL-6, pg/mL	1.02	0.98-1.07	0.30	0.50 (0.44-0.57)
IL-10, pg/mL	1.00	0.99-1.01	0.85	0.55 (0.49-0.61)
IL-17, pg/mL	1.01	0.98-1.05	0.51	0.53 (0.45-0.60)
MMP-2, ng/mL	1.00	0.95-1.05	0.96	0.51 (0.46-0.57)
MMP-9, ng/mL	1.05	0.99-1.11	0.12	0.55 (0.49-0.60)
MMP-2/MMP-9 ratio	1.01	0.98-1.03	0.58	0.55 (0.50-0.61)
TIMP-1, ng/mL	1.00	0.99-1.00	0.92	0.52 (0.44-0.60)
TIMP-4, ng/mL	1.00	0.99-1.00	0.67	0.51 (0.42-0.59)
Medications				
Carvedilol	1.15	0.70-1.89	0.58	0.51 (0.46-0.57)
ACE inhibitors/ARB	1.49	0.89-2.49	0.12	0.54 (0.48-0.59)
Digoxin	0.95	0.38-2.39	0.91	0.50 (0.45-0.56)
Spironolactone	1.35	0.71-2.59	0.36	0.52 (0.46-0.57)
Furosemide	1.09	0.59-2.01	0.79	0.50 (0.45-0.56)
Warfarin	1.59	0.84-3.03	0.15	0.52 (0.47-0.58)
Amiodarone	1.89	0.96-3.75	0.065	0.53 (0.48-0.59)
Metformin	0.77	0.44-1.35	0.37	0.52 (0.46-0.57)

ACE: angiotensin converting enzyme; ARB: angiotensin receptor blocker; AUC: area under the ROC curve; BNP: brain natriuretic peptide; CI: confidence interval; E: peak early wave diastolic filling velocity; E’: peak early diastolic mitral annulus velocity; IL: interleukin; LA: left atrial; LAHB: left anterior haemiblock; LBBB: left bundle branch block; LV: left ventricular; LVd: LV end-diastolic diameter; LVs: LV end-systolic diameter; MMP: matrix metalloproteinase; OR: odds ratio; RBBB: right bundle branch block; TGF-β1: transforming growth factor β1; TIMP: tissue inhibitor of metalloproteinases; TNF: tumoural necrosis factor.

Among biomarkers, the univariate analysis did not reveal any significant association with a positive *T. cruzi* PCR (Table II).

Other variables with a p value under 0.20 in the univariate analysis and which were included in the multivariate analysis were CD cardiac form, sex, body mass index, diabetes mellitus, LV aneurysm, MMP-9, use of either ACE inhibitor or ARB, use of warfarin, and use of amiodarone.

*Adjusted analysis between T. cruzi PCR positivity and studied variables* - Multivariate logistic regression analysis revealed that diabetes mellitus, right bundle branch block (RBBB), and history of previous trypanocidal treatment remained independently associated with a positive *T. cruzi* PCR with a p value < 0.0001 and an area under the ROC curve (AUC) of 0.65 (95% CI 0.60-0.70) for the overall model fit (Table III).

Among patients with a previous history of trypanocidal treatment, only four patients had a positive *T. cruzi* PCR and none of them received benznidazole 300 mg daily for at least 60 days: one patient received benznidazole 100 mg a day for 60 days; one patient was given benznidazole 200 mg a day for 60 days; one patient was treated with ketoconazole for 60 days; and one patient used benznidazole 300 mg a day for only 11 days due to adverse events.

**TABLE III:** Adjusted association between clinical and biomarker characteristics of studied subjects and *Trypanosoma cruzi* polymerase chain reaction (PCR) positivity

Variable	OR	95% CI	p values
Diabetes mellitus	0.53	0.30-0.93	0.028
History of trypanocidal treatment	0.13	0.04-0.38	0.0002
RBBB	1.78	1.10-2.89	0.019

Variables entered in the model: Chagas disease cardiac form, sex, body mass index, diabetes mellitus, history of trypanocidal treatment, right bundle branch block (RBBB), left ventricular (LV) aneurysm, matrix metalloproteinase 9 (MMP-9), use of either angiotensin converting enzyme (ACE) inhibitor or angiotensin receptor blocker (ARB), use of warfarin, and use of amiodarone. CI: confidence interval; OR: odds ratio.

*Regression analysis between parasite load and studied variables* - There was no significant univariate association between parasite load and age, body mass index, 2D echocardiographic parameters or biomarkers. For all analyses, residuals had a normal distribution (Table IV). A multiple regression analysis including variables with a p value under 0.20 on the univariate analysis did not retain any variable with significant association with the parasite load.

**TABLE IV:** Regression analysis associations between studied variables and parasite load

Variable	R^2^	p value
Age, years	0.00	0.79
Body mass index, kg/m^2^	0.00	0.80
2D echocardiogram		
LA volume, ml/m^2^	0.00	0.78
LVd, cm	0.00	0.72
LVs, cm	0.00	0.51
LV ejection fraction, %	0.01	0.31
E/E’ ratio	0.01	0.22
Biomarkers		
BNP, ng/mL	0.02	0.13
Troponin I, ng/mL	0.01	0.22
TGF-β1, ng/mL	0.00	0.96
TNF, pg/mL	0.01	0.31
IL-6, pg/mL	0.02	0.15
IL-10, pg/mL	0.02	0.15
IL-17, pg/mL	0.01	0.35
MMP-2, ng/mL	0.00	0.97
MMP-9, ng/mL	0.00	0.76
MMP-2/MMP-9 ratio	0.00	0.42
TIMP-1, ng/mL	0.01	0.32
TIMP-4, ng/mL	0.03	0.18

BNP: brain natriuretic peptide; E: peak early wave diastolic filling velocity; E’: peak early diastolic mitral annulus velocity; IL: interleukin; LA: left atrial; LV: left ventricular; LVd: LV end-diastolic diameter; LVs: LV end-systolic diameter; MMP: matrix metalloproteinase; TGF-β1: transforming growth factor β1; TIMP: tissue inhibitor of metalloproteinases; TNF: tumoural necrosis factor.

*Biomarkers* - As none of the biomarkers were associated with a positive *T. cruzi* PCR, we further investigated whether there were differences in the biomarker blood levels across the studied groups. For this analysis, patients with cardiac and cardiodigestive forms were grouped together, and the digestive form group was not included due to the low number of individuals in this group.

Brain natriuretic peptide (BNP), cardiac troponin I, matrix metalloproteinases 2 and 9 (MMP-2 and MMP-9), transforming growth factor β1 (TGF-β1), and tumour necrosis factor (TNF) blood levels were previously investigated in this project.^17^ IL-17 blood levels were higher in all cardiac form groups than in patients with the indeterminate form. IL-6 presented a tendency for higher blood levels in both initial stages of the cardiac form (p = 0.06) and advanced stages of the cardiac form (p = 0.05) than in patients with the indeterminate form. IL-10 and TIMP-1 blood levels were similar across the studied groups. TIMP-4 blood levels were higher in the initial stages of the cardiac form than in patients with the indeterminate form (Table V).

**TABLE V:** Biomarkers blood levels of studied subjects

		Cardiac form	
Variable	Indeterminate form n = 92	Stages A/B n = 179	Stages C/D n = 51
IL-6, pg/mL	2.5 (0.8-4.0)	3.2 (1.8-4.5)	3.6 (2.0-4.7)
IL-10, pg/mL	20.1 (16.2-23.1)	19.5 (9.4-23.0)	20.2 (18.3-22.8)
IL-17, pg/mL	5.0 (3.6-6.5)	5.7 (4.4-8.0)*	6.4 ± 3.0*
TIMP-1, ng/mL	0.39 **±** 0.18	0.37 **±** 0.16	0.41 **±** 0.22
TIMP-4, ng/mL	0.62 **±** 0.39	0.81 **±** 0.47*	0.82 **±** 0.64

IL: interleukin; TIMP: tissue inhibitor of metalloproteinases. Values are mean ± standard deviation or median (interquartile range). *p < 0.05 vs. indeterminate form.

## DISCUSSION

Parasite persistence in *T. cruzi* chronic infection plays a central role in CD progression and reactivation. However, the mechanisms associated with the development of CD pathology are still not fully clarified and it has been suggested that alterations in the immune response profile are involved in the pathogenesis of the disease. Previous data have already reported a possible association between cytokine levels and individuals in the indeterminate and cardiac CD clinical forms.^24^ ^,^ ^25^ An imbalance between pro-inflammatory and anti-inflammatory networks has been suggested as being important in CD progression towards Chagas heart disease.^12^ ^,^ ^13^

However, we could not demonstrate an association between biomarker blood levels and persistent parasitaemia. The strongest association with a negative *T. cruzi* PCR was previous history of trypanocidal treatment indicating the high anti-parasite efficiency of benznidazole, as patients remained negative even decades after treatment. The other two factors associated with *T. cruzi* PCR were the presence of RBBB and diabetes mellitus.

Cure criteria after anti-parasite treatment are currently limited to seroconversion. However, the change in serological status (seronegativity using conventional serological tests) may take decades to happen.^26^ Therefore, new cure criteria are needed in order to follow patients properly and test new treatment strategies. Thus, biomarkers before and after trypanocidal treatment have been studied and some have been shown to present significant changes after trypanocidal treatment. Those include the sera reactivity for a set of *T. cruzi* antigens (KMP11, PFR2, HSP70, and 3973d)^27^ and a specific antibody response (antibody 3, Ab3)^28^ that declined significantly in BZN-treated patients, and a change in antigen-specific response of CD8 T cells with an improved response after trypanocidal treatment.^29^ This may be relevant, as dysfunction of CD8 T cells may contribute to an immunological imbalance that compromises pathogen control in CD.^30^

Regarding *T. cruzi* PCR, a persistent positive test after trypanocidal treatment is recognised as a treatment failure. There is also an association between a drop of serological reactivity and a decrease of *T. cruzi* PCR positivity.^31^ Yet another important aspect is that *T. cruzi* PCR positivity is associated with Chagas heart disease^32^ and worse clinical prognosis.^17^ However, a negative test cannot be used as cure criterion, as parasitaemia is low and intermittent in chronic CD. Nonetheless, it is the best parameter to be followed in clinical trials testing new treatment strategies against CD.^33^ Therefore, we evaluated the association between a positive *T. cruzi* PCR and several biomarkers described to have changes in their blood levels in patients with CD. However, we could not find such a correlation even though we have shown changes in cytokine blood levels in CD clinical forms, similar to the literature. We showed that IL-17 blood levels were higher in all cardiac form groups than in patients with the indeterminate form, as shown by others.^34^ IL-6 presented a tendency for higher blood levels in both the initial stages of the cardiac form and advanced stages of the cardiac form than in patients with the indeterminate form, as shown by others.^14^ ^,^ ^34^ ^,^ ^35^ Additionally, a specific IL6 genotype lowered the risk of positive parasitaemia.^36^ We have also shown previously that the group of patients studied in this article had changes in BNP, cardiac troponin, TNF and TGF-β1 in patients with the cardiac form like what is described in the literature, and discussed these findings.^17^

We found that the presence of diabetes mellitus was a protective factor regarding *T. cruzi* PCR positivity. This could be interpreted as a contradictory finding, as hyperglycaemia was shown to increase parasitaemia and mortality in an animal model of acute CD^37^ and patients with diabetes mellitus are known to have increased susceptibility to infection, which is linked to immune dysfunction.^38^ However, the main drug used to treat diabetes patients included in this study (metformin) was recently shown to have an *in vitro* parasite-killing activity against *T. cruzi* thought to be mediated by the blocking of the mitochondrial respiratory-chain complex I.^39^ In fact, biguanides were first developed to be used as an antimalarial drug, but their effect on lowering blood glucose levels resulted in their use as a drug to treat diabetes mellitus.^40^ Therefore, it is possible that the chronic use of metformin, among other factors, could have contributed to a decreased risk of *T. cruzi* PCR positivity present by patients with diabetes mellitus.

The presence of RBBB was the only characteristic evaluated by cardiac exams found to be associated with a positive *T. cruzi* PCR. In fact, the frequency of a positive *T. cruzi* PCR or the parasite load was similar across patients with different forms of the chronic CD. We can speculate that the predilection of the *T. cruzi* for the heart conduction system^41^ may be linked to the higher risk of a positive *T. cruzi* PCR among those with a RBBB. Previous work has described that patients with low parasitaemia had a significantly higher risk of having a reduced LV ejection fraction than patients with undetectable parasitaemia, although those with a positive *T. cruzi* PCR had a lower ejection fraction.^16^

*Clinical implications* - Although there are many biomarkers being evaluated as cure criteria, none of the biomarkers studied here, including some with prognostic association in previous studies, were found to be associated with the presence of a positive or negative *T. cruzi* PCR. On the other hand, the strongest factor associated with a negative *T. cruzi* PCR was previous history of trypanocidal treatment with benznidazole, especially in those patients who completed treatment with a dose of 5 mg/kg/day. It is noteworthy that patients within this article were treated for an average of 11 years before the PCR evaluation, which shows a long-term efficacy of the benznidazole treatment. This reinforces the importance of the trypanocidal treatment.

*Limitations* - This work is limited by the retrospective nature of the data collected on previous history of trypanocidal treatment. Also, parasite load is low and has a skewed distribution, which limits the study of its association with clinical characteristics.

*In conclusion* - Among several clinical and biomarkers parameters, only diabetes mellitus, right bundle branch block, and history of trypanocidal treatment showed an independent association with *T. cruzi* PCR. History of trypanocidal treatment was a strong protective factor against a positive *T. cruzi* PCR even more than 10 years after treatment.
